# Supplementary material for: The Acute Phase Reaction and Its Prognostic Impact in Patients with Head and Neck Squamous Cell Carcinoma: Single Biomarkers Including C-Reactive Protein Versus Biomarker Profiles
Source: Biomedicines. 2020 Oct 14;8(10):418. doi: 10.3390/biomedicines8100418 (PMC7602291; doi:10.3390/biomedicines8100418)
Supplement: Supplementary file 1 [file biomedicines-08-00418-s001.pdf]

SUPPLEMENTARY INFORMATION

The Acute Phase Reaction and Its Prognostic Impact in Patients with Head and Neck Squamous Cell Carcinoma: Single Biomarkers Including C-Reactive Protein versus Biomarker Profiles

Table S1. Clinical and biological characteristics of the 144 patients with head and neck squamous cell carcinoma at the time of first diagnosis.

PARAMETER	VALUE
**Patients**	
Age (median and range, years)	62.0 (37-82)
Gender (males/females, numbers)	105/39
**Blood values (median and range)**[1]	
Hemoglobin (g/100 mL)	14.7 (9.7-17.7)
Leukocytes (x 10^9/L)	7.0 (4.0-21.1)
Thrombocytes (x 10^9/L)	255 (112-759)
CRP (mg/L)	3 (1-150)
Erythrocyte sedimentation rate (mm/hour)	14 (1-106)
Serum-glucose (mM)	5.8 (3.5-15.2)
Serum-creatinine (μM)	77 (47-167)
**Cancer characteristic**	
HPV status (negative/positive; missing in 42 patients)	45/57
Primary site of the tumor	
Tonsils, tongue base, head and neck cancer origo inserta	55 (38.2)
Other oropharyngeal sites	16 (11.1)
Oral cavity	62 (43.1)
Other (nasopharynx, hypopharynx, glottis)	11 (7.6)
T stage (number and percentage)	
T0/unknown primary	7 (4.9)
T1	42 (29.2)
T2	63 (43.8)
T3	13 (9.0)
T4	19 (13.2)
N stage (number and percentage)[2]	
N0	68 (47.2)
N1	22 (15.3)
N2	51 (35.4)
N3	3 (2.1)
M stage	All patients M0
**Primary treatment**	
Surgery including neck dissection (yes/no, numbers)[3]	Primary surgery 74/70 patients<br>Neck dissection 42/102 patients
Radiotherapy (yes/no, numbers – 2 patients missing)[4]	118/26 patients
Chemotherapy (yes/no, number of patients – missing data for one patient)[4]	69/75 patients

[1] Erythrocyte sedimentation rate was available for 139 patients and glucose levels for 141 patients; the other data were available for 144 patients.
[2] Lymph node metastasis according to the Union for International Cancer Control, seventh edition (2009)
[3] Surgical treatment was either resection of the primary tumor, resection of the primary tumor combined with neck dissection, or neck dissection only.
[4] Other treatment options in the curative setting are radio- and/or chemotherapy.

Table S2. An overview of the tumor and nodal staging for the 144 head and neck squamous cell carcinoma patients included in the study. The results are presented as the number of patients. The grey shadow indicates patients referred to as patients with less advanced disease, all other patients were classified as advanced disease, i.e., having either a locally advanced tumor or lymph node affection.

		**N stage**				
		**0**	**1**	**2**	**3**	**Total**
**T stage**	**0**	0	1	5	1	7
	**1**	30	4	8	0	42
	**2**	27	12	22	2	63
	**3**	4	1	8	0	13
	**4**	7	4	8	0	19
	**Total**	68	22	51	3	144

Table S3. Plasma levels of IL6 family members, IL1 subfamily members, and TNFα in patients with head and neck squamous cell carcinoma (HNSCC); a comparison of the cancer patients with healthy controls. IL6 levels were determined for all 144 patients. Due to technical reasons, cytokine levels were only available for 143 patients except for CNTF levels that were available for 142 patients. All concentration results are presented as the median and variation range. The Mann-Whitney U-test was used for the statistical comparisons. The presentation to the right in the table shows the number of patients with levels below, within or above the corresponding variation range for the healthy controls.

	Mediator concentration (pg/ml)			Patient levels compared with the variation range for healthy controls (number)		
**Mediator**	**HNSCC**	**Healthy controls (n = 15)**	**p-value**	**Below**	**Within**	**Above**
gp130	82,014 (25,184-108,957)	74,049 (33,245-91,488)	0.187	1	112	30
IL6Rα	32,860 (5119-47,290)	32,501 (21,885-36,651)	0.207	3	98	42
IL6	↑ 1.87 (0.20-45.03)	0.67 (0.27-1.34)	**< 0.001**	1	39	104
IL27	↑ 457 (116-1096)	387 (233-1058)	**0.029**	10	132	1
IL31	↑ 71.2 (19.4-132.2)	51.4 (23.4-73.7)	**0.005**	5	78	60
OSM	4603 (2487-5799)	4208 (3290-5345)	0.058	4	127	12
CNTF	↓ 577 (33.8-13,595)	1980 (179-5648)	**0.012**	15	120	7
IL1RA	↑ 382 (119-3185)	268 (199-677)	**0.007**	4	111	28
IL33Rα	17,520 (75.9-113,011)	14,309 (8256-35,541)	0.087	6	124	13
TNFα	↑ 16.5 (2.6-28.6)	13.3 (1.1-20.9)	**0.018**	0	110	33

Table S4. Hierarchical clustering analysis based on plasma levels of seven IL6 family members in head and neck squamous cell carcinoma patients; a comparison of the mediator levels in each of the two main clusters with the levels in healthy controls. Plasma levels of seven IL6 family members, two IL1 subfamily members, and TNFα in patients with head and neck squamous cell carcinoma were compared with a group of healthy controls for the two main patient clusters that were identified by unsupervised hierarchical clustering analysis based on seven IL6 family members (Figure 1). All results are presented as the median and the variation range. The Mann-Whitney U-test was used for the statistical comparisons. Due to technical reasons, cytokines/receptor levels were only available for 143 (99/44) patients, CNTF levels were available for 142 (98/44) patients, whereas IL6 levels were determined for all 144 patients.

Mediator (pg/mL)	Healthy controls (n = 15)	Upper IL6 family cluster (n = 99)	p-value	Lower IL6 family cluster (n = 45)	p-value
gp130	74,049 (33,245-91,488)	81,943 (25,184-108,957)	0.260	83,253 (41,057-101,998)	0.126
IL6Rα	32,501 (21,885-36,651)	32,289 (5119-44,259)	0.428	↑ 34,839 (21,527-47,290)	**0.040**
IL6	0.67 (0.27-1.34)	↑ 1.66 (0.20-9.41)	**< 0.001**	↑ 2.50 (0.83-45.03)	**< 0.001**
IL27	387 (233-1058)	457 (116-1096)	0.055	↑ 463 (116-882)	**0.015**
IL31	51.4 (23.4-73.7)	↑ 62.5 (19.4-132.2)	**0.025**	↑ 78.5 (23.8-123.0)	**< 0.001**
OSM	4208 (3290-5345)	4642 (2487-5799)	0.065	4531 (3165-5747)	0.086
CNTF	1980 (179-5648)	1113 (293-13,595)	0.251	↓ 206 (33.8-1980)	**< 0.001**
IL33Rα	14,309 (8256-35,541)	16,950 (75.9-107,876)	0.178	↑ 18,588 (8434-113,011)	**0.026**
IL1RA	268 (199-677)	↑ 356 (119-3185)	**0.023**	↑ 468 (194-1409)	**0.001**
TNFα	13.3 (1.1-20.9)	15.8 (2.6-28.6)	0.071	↑ 17.5 (2.6-28.6)	**0.001**

Table S5. Hierarchical clustering analysis based on plasma levels of seven IL6 family members, two IL1 subfamily members (IL1RA, IL33Rα) and TNFα; a comparison of the two main clusters with a group of healthy controls. Plasma levels of IL6 family members, IL1 subfamily members, and TNFα in patients with head and neck squamous cell carcinoma; a comparison of levels for each of the two main clusters that were identified by unsupervised hierarchical clustering analysis based on all 10 soluble mediators included in our study (Figure 2), and the group of healthy controls. All results are presented as the median and the variation range. The Mann-Whitney U-test was used for the statistical comparisons. Due to technical reasons, mediator levels were only available for 143 (66/77) patients except for CNTF levels that were available for 142 (65/77) patients. IL6 levels were measured for all 144 patients.

**Mediator (pg/mL)**	**Healthy controls (n = 15)**	**Upper main cluster * (n = 66)**	**p-value**	**Lower main cluster (n = 77)**	**p-value**
gp130	74,049 (33,245-91,488)	76,526 (40,175-108,957)	0.584	83,538 (25,184-107,124)	0.061
IL6Rα	32,501 (21,885-36,651)	31,413 (5119-44,523)	0.932	↑ 34,288 (21,943-47,290)	**0.030**
IL6	0.67 (0.27-1.34)	↑ 2.10 (0.20-45.03)	**< 0.001**	↑ 1.80 (0.36-10.00)	**< 0.001**
IL27	387 (233-1058)	415 (116-1096)	0.327	↑ 485 (116-882)	**0.002**
IL31	51.4 (23.4-73.7)	62.5 (19.4-132.2)	0.156	↑ 75.7 (23.8-123.0)	**< 0.001**
OSM	4208 (3290-5345)	4647 (2487-5799)	0.081	4603 (3165-5747)	0.064
CNTF	1980 (179-5648)	1980 (293-13,595)	0.650	↓ 312 (33.8-881)	**< 0.001**
IL33Rα	14,309 (8256-35,541)	17,359 (75.9-113,011)	0.224	↑ 18,202 (7462-64,650)	**0.046**
IL1RA	268 (199-677)	↑ 400 (119-3185)	**0.008**	↑ 361 (194-1152)	**0.012**
TNFα	13.3 (1.1-20.9)	14.4 (2.6-26.2)	0.310	↑ 18.2 (2.6-28.6)	**0.001**

* One additional patient was allocated to this group due to relatively high CNTF

Table S6. Plasma levels of IL6 family members, IL1 subfamily members, TNFα, and CRP in patients with head and neck squamous cell carcinoma; a comparison of HPV-negative (i.e., patients tested to be negative or assumed to be negative due to the tumor site) and HPV-positive patients. All results are presented as the median and the variation range. The Mann-Whitney U-test was used for the statistical comparisons. Due to technical reasons, mediators were only available for 143 (86/57) patients except for CNTF levels that were available for 142 (85/57) patients. IL6 and CRP was analyzed in all 144 patients.

**Mediator**	**HPV-negative or unkown (n = 87)****	**HPV-positive (n = 57)**	**p-value**
gp130 (pg/mL), n = 143 (86/57)	82,495 (40,175-108,957)	80,566 (25,184-101,998)	0.335
IL6Rα (pg/mL), n = 143 (86/57)	31,554 (5119-47,290)	35,037 (21,527-44,259)	**0.001**
IL6 (pg/mL), n = 144 (87/57)	2.09 (0.20-15.91)	1.71 (0.40-45.03)	0.121
IL27 (pg/mL), n = 143 (86/57)	457 (116-1096)	463 (116-714)	0.632
IL31 (pg/mL), n = 143 (86/57)	63.6 (19.4-132.2)	75.7 (19.4-117.4)	0.056
OSM (pg/mL), n = 143 (86/57)	4521 (2487-5799)	4749 (3516-5508)	0.160
CNTF (pg/mL), n = 142 (85/57)	577 (33.8-9864)	577 (33.8-13,595)	0.467
IL33Rα (pg/mL), n = 143 (86/57)	17,701 (75.9-107,876)	17,520 (3480-113,011)	0.837
IL1RA (pg/mL), n = 143 (86/57)	373 (119-3185)	395 (193-2187)	0.711
TNFα (pg/mL), n = 143 (86/57)	15.8 (2.9-28.6)	17.3 (2.6-28.6)	0.351
CRP (mg/L), n = 144 (87/57)	3 (1-150)	3 (1-54)	0.657

** All primary tumors except one in 42 patients with undetermined HPV-status were located outside the oropharynx. For this reason, the tumors were designated as HPV-negative. The remaining 45 to the total of 87 patients was tested to be negative.

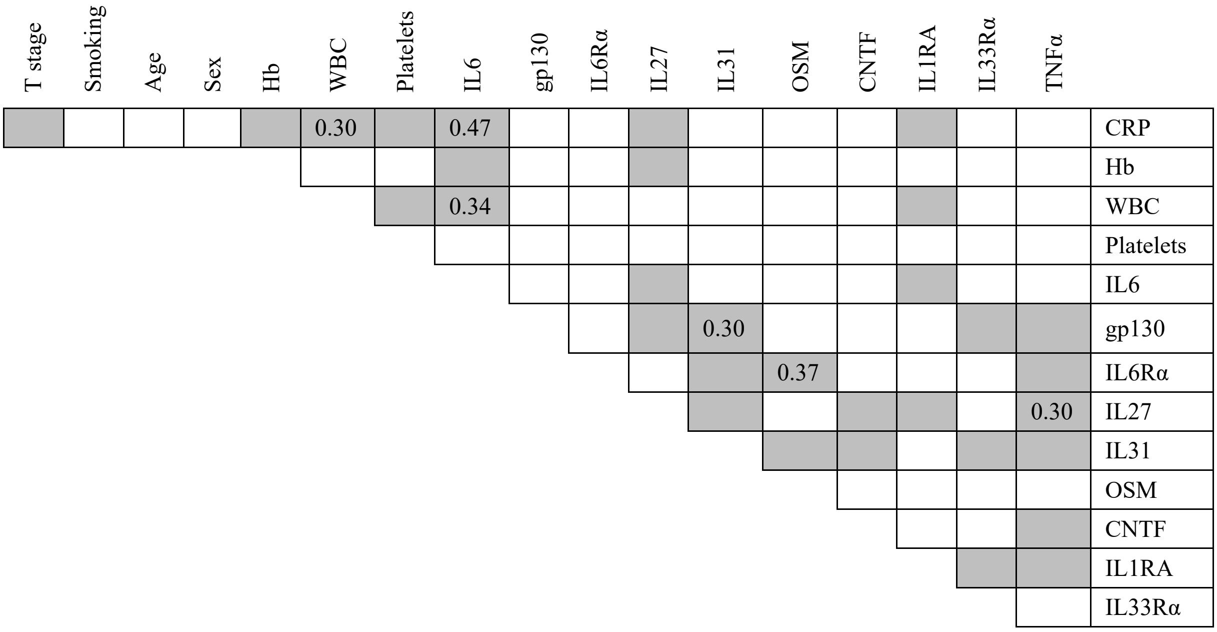

Figure S1. Correlation analysis of clinical parameters and inflammatory markers in head and neck squamous cell carcinoma; a study of 144 consecutive patients. The study included CRP level, hemoglobin (Hb) level, white blood cell (WBC) and platelet counts in peripheral blood and soluble plasma levels of IL6 family (gp130, IL6Rα, IL6, IL27, IL31, OSM, CNTF), IL1 subfamily (IL1RA, IL33Rα) and TNFα. Correlations with a p-value below 0.01 are indicated by grey shadow, correlation coefficients ≥ 0.30 are also indicated in the figure.

Death	HPV	Age	Smoking	T status	N status
0	0	64	4	2	2
1	3	64	4	4	2
0	3	62	4	1	0
1	1	78	4	2	2
0	3	79	4	2	0
0	3	37	2	2	0
0	3	78	0	2	0
0	3	70	4	1	0
0	1	52	0	3	2
0	1	62	1	1	2
1	1	71	2	4	2
1	0	62	2	2	2
2	0	82	4	2	0
0	0	68	4	1	0
1	0	59	4	3	2
0	3	70	4	1	0
0	0	41	3	3	0
0	1	42	4	2	2
0	0	56	0	2	0
0	1	55	0	1	2
0	3	75	0	3	0
1	0	44	4	4	2
0	1	67	3	4	0
0	2	61	0	2	1
0	0	58	4	2	0
1	0	64	4	4	2
1	0	51	0	3	2
2	0	70	3	0	2
0	1	63	4	2	2
0	3	56	0	2	1
0	2	58	0	2	0
0	0	43	2	2	0
0	1	56	0	3	2
0	0	51	4	0	2
0	3	47	1	2	0
0	0	39	0	2	0
0	3	75	0	1	0
0	1	57	1	0	2
0	1	50	0	2	2
0	1	51	4	2	2
0	1	54	4	3	2
0	0	56	4	1	0
0	1	67	0	2	1
0	3	49	4	2	0
0	0	59	4	2	0
1	2	69	4	4	2
0	1	45	1	1	2
0	1	71	0	0	2
0	3	64	4	1	0
0	1	52	0	2	0
0	0	68	4	1	0
0	3	65	4	4	0
0	3	72	4	1	0
0	0	71	0	2	0
1	3	66	4	4	0
0	0	69	4	0	2
0	1	53	4	2	1
0	0	70	3	2	0
0	3	67	4	2	0
0	3	77	0	4	0
0	0	72	4	1	0
0	1	68	4	2	1
0	3	64	4	1	0
1	3	63	2	4	2
1	3	61	4	2	2
0	0	75	4	2	0
0	3	62	4	1	0
0	1	53	2	1	1
0	1	70	2	4	1
0	1	62	0	2	2
0	3	46	4	1	0
0	0	47	0	1	0
0	3	66	0	1	0

Death:

0	Still alive
1	Death from the cancer
2	Death from other causes

HPV status:

0	HPV-negative
1	HPV16-positive
2	Other HPV-positive
3	Designated HPV-negative

Age (years given in the corresponding figure column):

	Below 70 years of age
	Aged 70 years or above

Smoking:

0	Never smoked
1	Less than 10 pack-years
2	Probably less than 10 pack-years
3	Probably more than 10 pack-years
4	More than 10 pack-years

T stage:

0	T0 – No evidence of primary tumor
1	T1 – Tumor 2 cm or less in greatest dimension
2	T2 – Tumor more than 4 cm in greatest dimension
3	T3 – Tumor more than 4 cm in greatest dimension
4	T4 – Advanced local disease (invasion into neighboring tissue/structures/spaces)

N stage:

0	N0 – No regional node metastasis
1	N1 – Single lymph node metastasis ≤ 3 cm (ipsilateral)
2	N2 – Ipsilateral lymph node metastasis up to 6 cm or any bi-/and contralateral
3	N3 – Lymph node metastasis > 6 cm

0	0	67	3	1	1
0	1	55	3	2	2
0	1	57	4	1	2
0	2	62	0	1	0
0	0	61	4	2	1
2	1	73	0	2	2
1	0	67	2	2	0
0	0	69	4	3	1
0	1	59	3	2	0
0	1	54	4	2	0
0	3	57	4	2	0
0	0	71	4	1	0
0	1	62	3	0	3
0	2	54	4	4	0
0	3	66	2	2	0
0	0	71	4	1	0
0	3	44	4	1	0
0	0	58	0	2	0
0	1	43	2	2	2
0	3	70		1	0
0	1	56	4	2	2
0	3	55		1	0
0	0	55	0	1	0
0	1	54	0	1	1
0	3	42	0	1	0
1	0	69	4	2	2

0	0	68	4	4	0
0	3	67	4	1	0
1	3	68	4	2	2
0	0	72	4	4	1
0	2	56	4	2	1
0	1	52	2	1	2
0	1	57	0	2	2
1	3	74	4	4	2
0	1	51	1	3	2
0	3	75	2	1	0
0	1	44	0	1	1
0	1	48	1	2	2
0	1	55	1	1	2
0	0	72	4	2	2
0	1	49	0	2	1
0	1	72	0	2	0
0	3	63	0	2	0
0	1	38	0	2	2
0	1	57	1	2	1
0	1	54	0	1	0
0	0	70	0	4	2
0	0	60	0	3	0
0	0	68	2	1	0
1	0	62	4	2	1
2	3	67	4	2	0
0	1	64	0	3	0
1	3	64	4	1	2
1	0	63	4	4	1
0	2	67	4	2	2
1	3	56	4	3	2
1	0	69	4	3	2
0	1	50	0	2	3
0	3	79	0	2	1
0	0	47	0	1	0
0	2	62	0	1	2
0	1	67	0	4	1
0	3	68	0	2	0
0	0	40	0	2	1
0	1	76	2	1	0
0	1	45	0	2	2
0	1	38	4	2	2
2	3	69	3	1	0
0	3	68	4	4	0
1	0	70	4	2	3
0	2	72	4	0	1

Figure S2. Unsupervised hierarchical clustering of HNSCC patients based on the plasma levels of IL6 family mediators; an overview of clinical and biological characteristics of the individual patients in the two main clusters (i.e., upper/left main cluster including the 99 patients above the red line; lower/right main cluster including the 45 patients below the red line).

Death	HPV	Age	Smoking	T status	N status
0	0	64	4	2	2
1	3	64	4	4	2
0	3	62	4	1	0
2	0	82	4	2	0
1	0	59	4	3	2
0	0	51	4	0	2
0	0	39	0	2	0
0	0	68	4	1	0
0	3	79	4	2	0
0	3	37	2	2	0
0	1	62	1	1	2
1	0	62	2	2	2
0	3	75	0	1	0
0	3	75	0	3	0
1	0	44	4	4	2
0	3	77	0	4	0
0	0	72	4	1	0
0	1	70	2	4	1
0	1	68	4	2	1
0	1	76	2	1	0
0	0	68	4	4	0
0	3	67	4	1	0
1	3	68	4	2	2
0	0	72	4	4	1
1	1	78	4	2	2
0	3	64	4	1	0
0	3	70	4	1	0
0	1	52	0	3	2
1	1	71	2	4	2
1	3	61	4	2	2
1	3	63	2	4	2
0	0	75	4	2	0
0	1	67	3	4	0
0	2	61	0	2	1
1	0	51	0	3	2
1	0	64	4	4	2
2	0	70	3	0	2
0	0	58	4	2	0
0	2	58	0	2	0
0	1	63	4	2	2
0	3	78	0	2	0
0	0	56	0	2	0
1	2	69	4	4	2
0	3	70	4	1	0
0	0	41	3	3	0
0	1	45	1	1	2
0	1	42	4	2	2
0	0	59	4	2	0
0	1	55	0	1	2
0	3	56	0	2	1
0	1	71	0	0	2
0	0	43	2	2	0
0	1	56	0	3	2
0	1	57	1	0	2
0	3	47	1	2	0
0	1	54	4	3	2
0	1	51	4	2	2
0	0	56	4	1	0
0	1	67	0	2	1
0	1	50	0	2	2
0	3	42	0	1	0
1	0	69	4	2	2
0	0	70	3	2	0
0	3	46	4	1	0
0	3	62	4	1	0
0	2	56	4	2	1
0	3	64	4	1	0
0	1	57	1	2	1
1	0	67	2	2	0
0	1	52	0	2	0
0	3	65	4	4	0
0	1	54	0	1	0
1	3	74	4	4	2
0	1	51	1	3	2

Death:

0	Still alive
1	Death from the cancer
2	Death from other causes

HPV status:

0	HPV-negative
1	HPV16-positive
2	Other HPV-positive
3	Designated HPV-negative

Age (years given in the corresponding figure column):

	Below 70 years of age
	Aged 70 years or above

Smoking:

0	Never smoked
1	Less than 10 pack-years
2	Probably less than 10 pack-years
3	Probably more than 10 pack-years
4	More than 10 pack-years

T stage:

0	T0 – No evidence of primary tumor
1	T1 – Tumor 2 cm or less in greatest dimension
2	T2 – Tumor more than 4 cm in greatest dimension
3	T3 – Tumor more than 4 cm in greatest dimension
4	T4 – Advanced local disease<br>(invasion into neighboring tissue/structures/spaces)

N stage:

0	N0 – No regional node metastasis
1	N1 – Single lymph node metastasis ≤ 3 cm (ipsilateral)
2	N2 – Ipsilateral lymph node metastasis up to 6 cm or any bi-/and contralateral
3	N3 – Lymph node metastasis > 6 cm

0	0	61	4	2	1
2	1	73	0	2	2
0	2	54	4	4	0
0	1	52	2	1	2
0	3	72	4	1	0
1	3	66	4	4	0
0	1	44	0	1	1
0	1	57	0	2	2
0	0	60	0	3	0
0	1	67	0	4	1
0	3	68	0	2	0
0	3	44	4	1	0
0	1	55	3	2	2
0	1	57	4	1	2
0	1	72	0	2	0
0	2	62	0	1	0
0	1	55	1	1	2
0	3	63	0	2	0
0	3	66	2	2	0
0	0	71	4	1	0
0	0	69	4	0	2
0	1	62	3	0	3
0	0	58	0	2	0
0	1	43	2	2	2
0	3	70		1	0
0	0	69	4	3	1
0	1	59	3	2	0
0	1	54	4	2	0
0	3	57	4	2	0
0	0	71	4	1	0
0	1	53	2	1	1
0	1	53	4	2	1
0	3	67	4	2	0
0	0	68	4	1	0
1	0	62	4	2	1
2	3	67	4	2	0
1	0	69	4	3	2
0	0	47	0	1	0
0	2	62	0	1	2
0	0	72	4	2	2
0	1	49	0	2	1
0	0	40	0	2	1
0	1	45	0	2	2
0	1	50	0	2	3
0	3	79	0	2	1
0	1	62	0	2	2
0	0	55	0	1	0
0	3	55		1	0
0	1	54	0	1	1
0	0	67	3	1	1
0	0	47	0	1	0
0	3	66	0	1	0
0	1	56	4	2	2
0	0	71	0	2	0
0	1	48	1	2	2
1	3	56	4	3	2
0	2	67	4	2	2
0	0	68	2	1	0
0	0	70	0	4	2
0	1	64	0	3	0
0	3	75	2	1	0
0	1	38	0	2	2
1	3	64	4	1	2
1	0	63	4	4	1
0	1	38	4	2	2
2	3	69	3	1	0
0	3	68	4	4	0
1	0	70	4	2	3
0	2	72	4	0	1
0	3	49	3	2	0

Figure S3. Unsupervised hierarchical clustering of HNSCC patients based on the plasma levels of six IL6 family mediators, two IL1 subfamily mediators and TNFα; an overview of clinical and biological characteristics of the individual patients in the two main clusters (i.e., upper main cluster including the 66 patients above the red line and the lowermost beneath the thinner; lower main cluster including the 77 patients below the thickest red line.

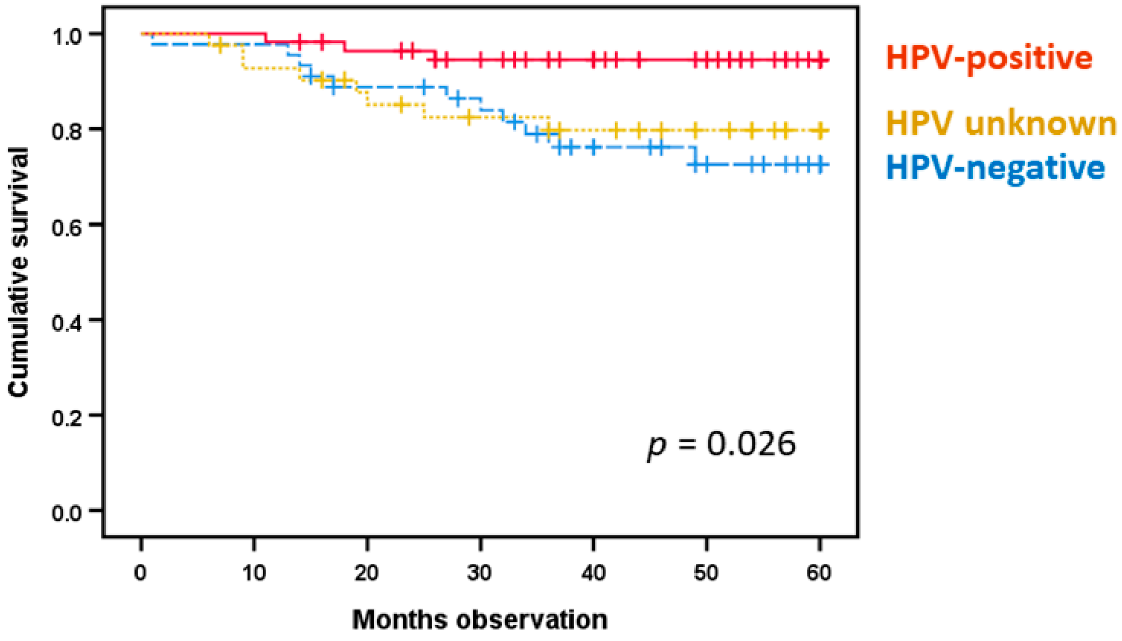

Figure S4. Comparison of head and neck cancer-related death according to human papilloma virus (HPV) tumor status. The upper red continuous line are patients with an HPV-positive tumor (n = 57). The lower blue semi-hatched line represents patients that have tested negative for HPV (n = 45), whereas the middle yellowish dotted line shows the survival of remaining patients, whose HPV-testing has not been performed due to site, i.e., located outside the oropharynx (n = 42). The p-value is presented in the figure. In further survival analyses, the two last groups (HPV-negative and unknown) were merged.
